# Supplementary material for: Retrieval and registration of long-range overlapping frames for scalable mosaicking of in vivo fetoscopy
Source: Int J Comput Assist Radiol Surg. 2018 Mar 15;13(5):713–20. doi: 10.1007/s11548-018-1728-4 (PMC5953985; doi:10.1007/s11548-018-1728-4)
Supplement: Supplementary file 1 — Supplementary material 1 (pdf 35 KB) [file 11548_2018_1728_MOESM1_ESM.pdf]

Supplementary material for IPCAI submission / IJCARS submission

Retrieval and Registration of Long-Range Overlapping Frames for Scalable Mosaicking of In Vivo Fetoscopy

The supplementary material consists of:

- **Files whose name starts with "Mosaicking_Videos":** Videos showing the creation of a mosaic by pasting frames of the sequence one by one on the canvas (such as the video used to generate Figure 3, as discussed in Section 3.2). Four mosaicking videos are available, respectively corresponding to the SURF-RANSAC baseline (Mosaicking_Videos_SURF_RANSAC.avi), the normalised cross-correlation baseline (Mosaicking_Videos_NCC.avi), our approach with purely sequential stiching (Mosaicking_Videos_Ours_Sequential.avi) and our best performing approach combining pairwise registrations with a retrieval of overlapping frames based on bag of words (Mosaicking_Videos_Ours_BoW.avi).

- **Files whose name starts with "Mosaics":** Images created by blending the aforementioned sequences (as shown in Figure 4). Note that, due to the poor performance of the baselines (especially NCC), the final visual rendering is not as informative as the videos above for NCC and SURF_RANSAC.

- **Files whose name starts with "Evaluation_Registrations":** Videos showing the manual labelling of pairwise registrations used to evaluate the registration approaches in Section 3.3 and generate the results in Table 1. Each video corresponds to one method and is structured as follows:
  - Frame #1: Frame #1 of the fetoscopy sequence
  - Frame #2: Frame #2 of the fetoscopy sequence registered and pasted on the previous frame
  - Frame #3: Frame #2 of the fetoscopy sequence
  - Frame #4: Frame #3 of the fetoscopy sequence registered and pasted on the previous frame

  and so on. For every even frame of the sequence, i.e. every frame where a registration result is shown, the color of the border of the video encodes the human annotation stating the estimated quality of the registration as follows:
  Green = Correct registration, Yellow = Doubtful, Red = Incorrect registration
