# Supplementary figures and images for: Retrieval and registration of long-range overlapping frames for scalable mosaicking of in vivo fetoscopy
Source: Int J Comput Assist Radiol Surg. 2018 Mar 15;13(5):713–20. doi: 10.1007/s11548-018-1728-4 (PMC5953985; doi:10.1007/s11548-018-1728-4)

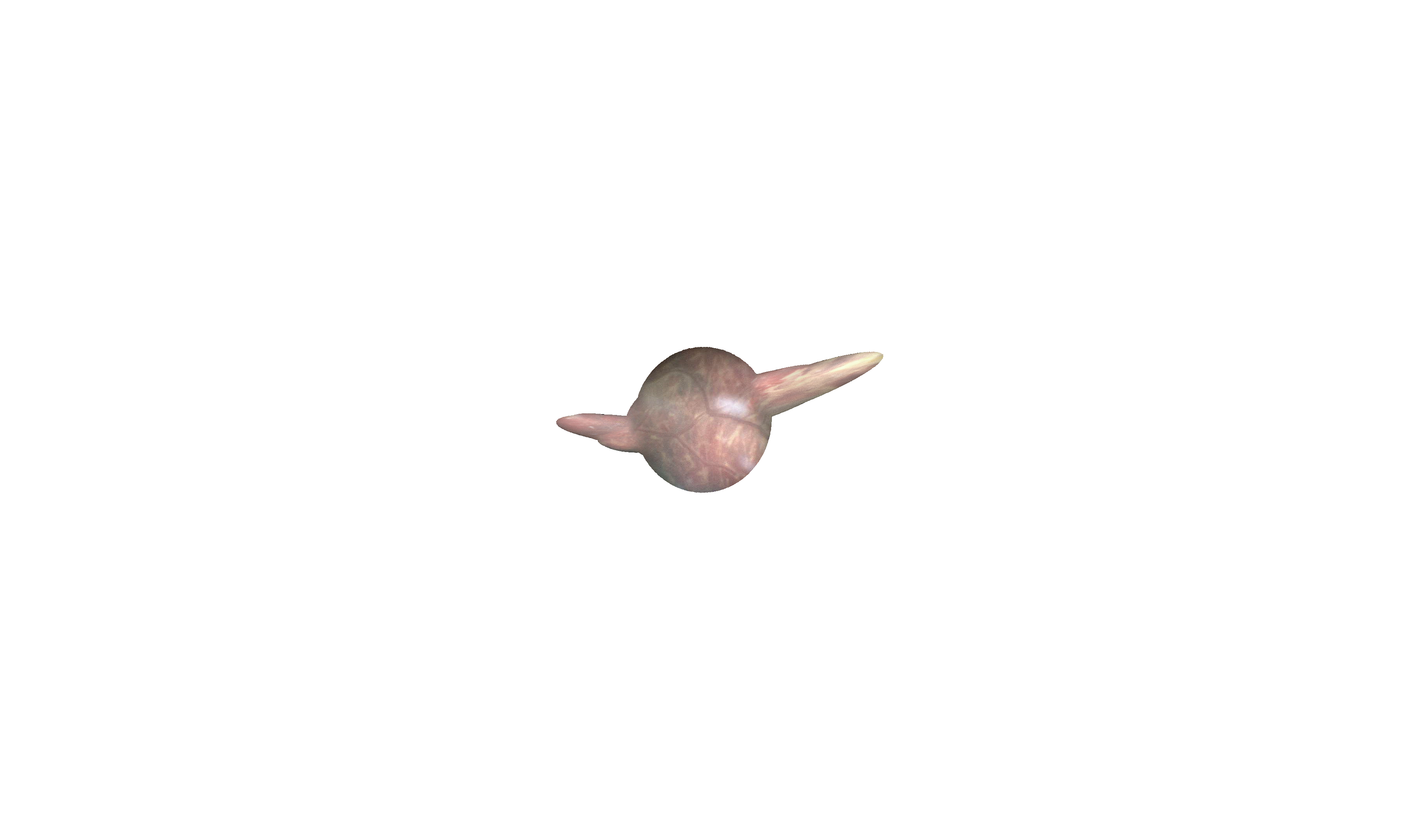

Supplement: Supplementary file 9 — Supplementary material 9 (png 324 KB) [file 11548_2018_1728_MOESM9_ESM.png]

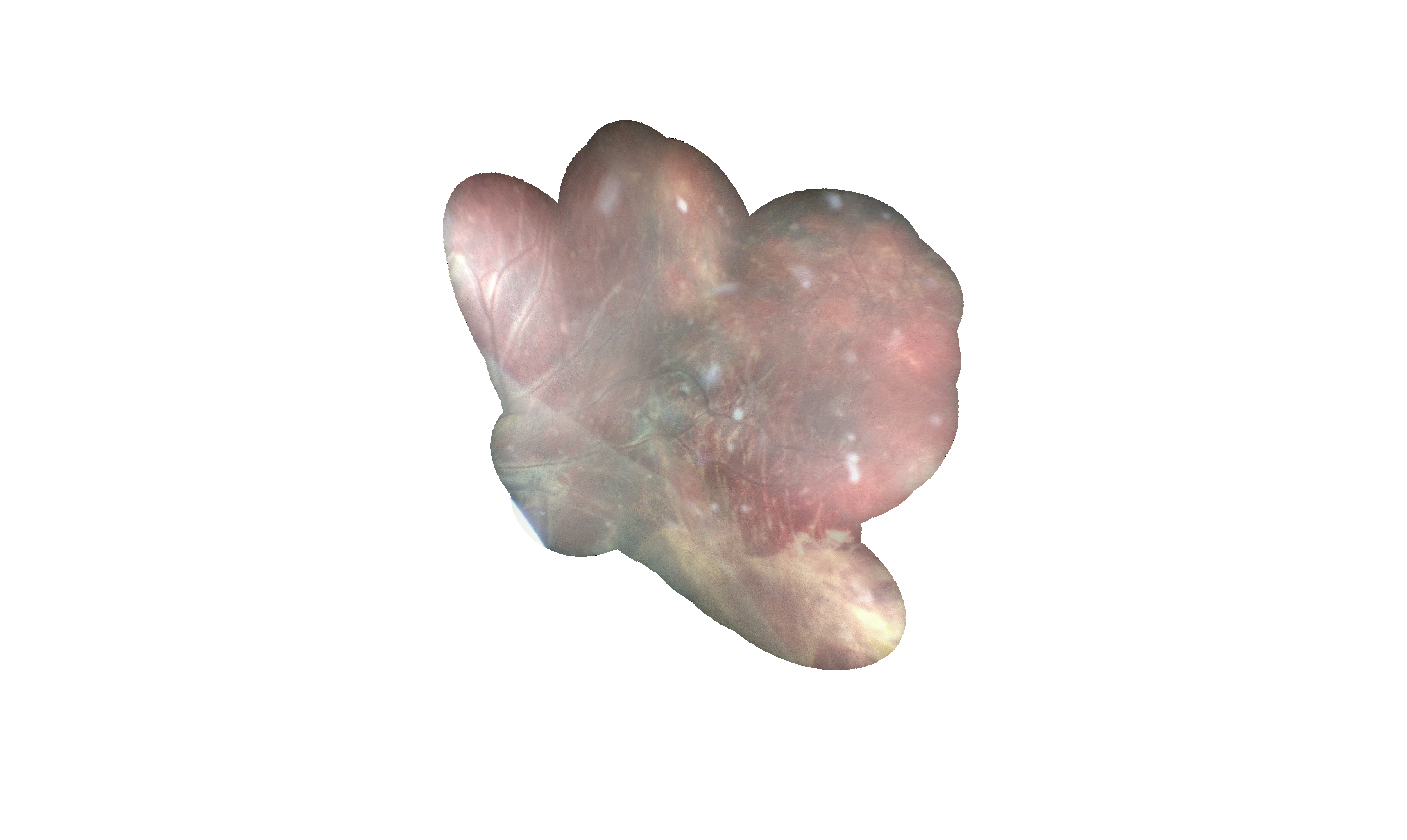

Supplement: Supplementary file 10 — Supplementary material 10 (png 2430 KB) [file 11548_2018_1728_MOESM10_ESM.png]

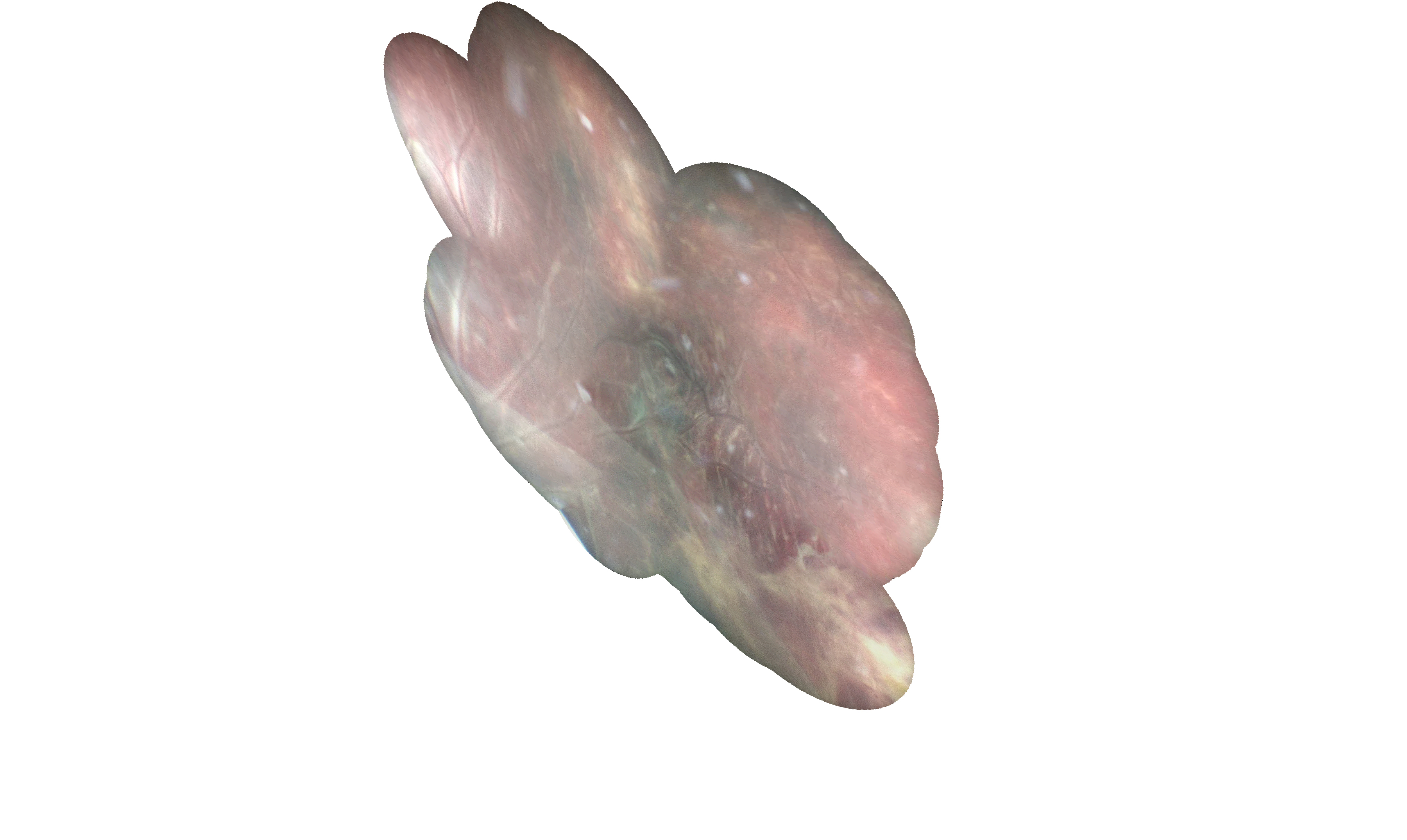

Supplement: Supplementary file 11 — Supplementary material 11 (png 2838 KB) [file 11548_2018_1728_MOESM11_ESM.png]

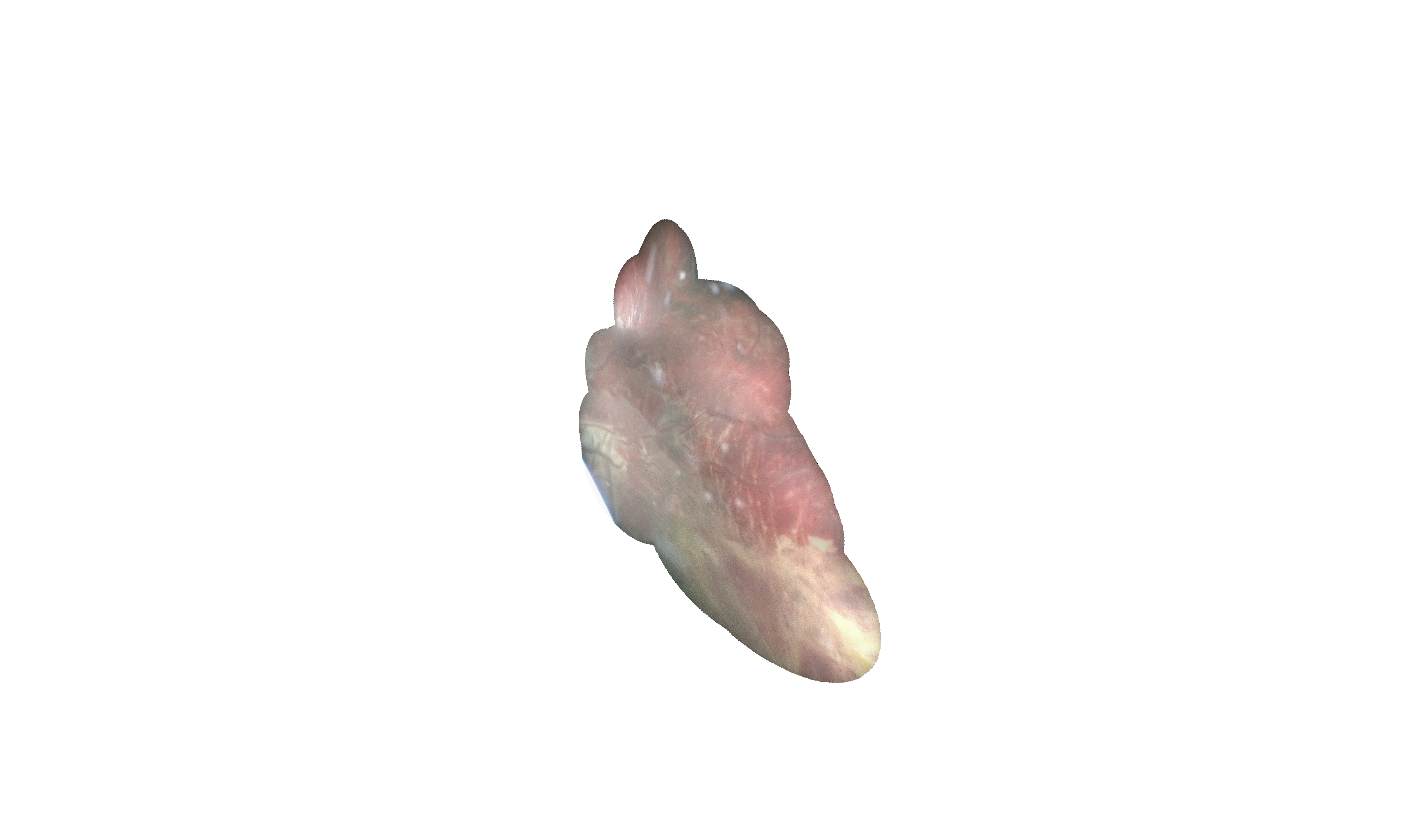

Supplement: Supplementary file 12 — Supplementary material 12 (png 1083 KB) [file 11548_2018_1728_MOESM12_ESM.png]
